# Impact of Infection Timing on Outcomes: A Comparative Study of Periprosthetic Joint and Fracture‐Related Infections

**DOI:** 10.1111/os.70323

**Published:** 2026-04-21

**Authors:** Lena Schwake, Jasper Frese, Arndt‐Peter Schulz, Sidney Schaeffer, Ulf‐Joachim Gerlach, Cornelius Grimme

**Affiliations:** ^1^ Section of Medicine Universität zu Lübeck Lübeck Germany; ^2^ Department of Septic Bone and Joint Surgery BG Klinikum Hamburg Hamburg Germany; ^3^ Center for Clinical Research BG Klinikum Hamburg Hamburg Germany

**Keywords:** fracture‐related infection, periprosthetic joint infection, quality of life, time factors, treatment outcome

## Abstract

**Objective:**

Implant‐associated infections, including periprosthetic joint infection (PJI) and fracture‐related infection (FRI), are among the most challenging complications in orthopedic surgery. Although infection timing (early, delayed, late) is recognized as an important factor in surgical success, its impact on patient‐reported outcomes and length of hospital stay has rarely been compared between PJI and FRI in a single cohort. This study aimed to examine the association between infection timing and health‐related quality of life (HRQoL) as well as length of hospital stay (LOS), and to explore potential differences between PJI and FRI.

**Methods:**

We conducted a retrospective monocentric cohort study of 60 patients with microbiologically confirmed implant‐associated infections treated at a German level‐I trauma center between January and December 2021, with follow‐up performed in 2023. Patients with PJI (*n* = 29) or FRI (*n* = 31) were stratified as early (< 3 months), delayed (3–24 months), or late (> 24 months) to enable direct comparison between infection entities. Outcomes included EQ‐VAS, EQ‐5D‐3L mean domain scores, LOS, reinfection, revision surgery, amputation, and in‐hospital mortality. Group differences were tested using independent‐samples t‐tests for continuous variables and chi‐square or Fisher's exact tests for categorical variables, with subgroup analyses considered exploratory due to small sample sizes.

**Results:**

Of the 60 patients, 25 had early (PJI *n* = 11; FRI *n* = 14), 24 delayed (PJI *n* = 11; FRI *n* = 13), and 11 late infections (PJI *n* = 7; FRI *n* = 4). EQ‐VAS was higher in FRI compared with PJI in early (63.4 ± 17.8, *n* = 13 vs. 48.3 ± 20.8, *n* = 9) and delayed infections (66.2 ± 16.8, *n* = 11 vs. 56.6 ± 13.5, *n* = 10), while both groups showed markedly lower EQ‐VAS values in late infections (FRI: 39.7 ± 25.9, *n* = 3 vs. PJI: 43.3 ± 23.4, *n* = 6), which were analyzed descriptively due to very small subgroup sizes. The EQ‐5D‐3L mean domain score was lower (indicating better health status) in early FRI (1.55 ± 0.33, *n* = 12) than in early PJI (1.91 ± 0.39, *n* = 9) in unadjusted analyses. LOS increased with timing, particularly in PJI (early 39.6 ± 26.8 vs. 31.4 ± 29.0 days; delayed 100.6 ± 82.2 vs. 28.2 ± 10.2; *p* = 0.015). Reinfection rates increased with later timing (early: 6/11 PJI vs. 3/14 FRI; delayed: 8/11 vs. 8/13; late: 5/7 vs. 4/4). Revision surgery was more frequent in early PJI (7/11 vs. 4/14; *p* = 0.080) but was significantly more common in delayed FRI (12/13 vs. 6/11; *p* = 0.033). Amputation (≤ 9%) and in‐hospital mortality (≤ 15%) were rare and showed no significant differences.

**Conclusions:**

Infection timing was associated with both clinical and patient‐reported outcomes in implant‐associated infections. Later infections were associated with poorer HRQoL, longer hospital stays, and higher reinfection rates, based on unadjusted analyses, with distinct patterns between PJI and FRI. Early recognition and timely, stage‐adapted treatment strategies may help improve patient outcomes.

## Graphical abstract

I commend the authors on their efforts in developing a strong manuscript centered around an important topic, the trajectory of health‐related quality of life following PJI. Their statistical analyses have been significantly improved, and their findings are both novel and clinically relevant. Their discussion is succinct, thorough, and readable. All results are interpreted appropriately. I have no additional concerns and recommend publication of this manuscript.

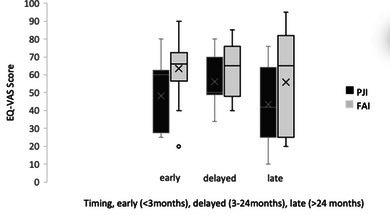

## 1 Introduction

Implant‐associated infections, including periprosthetic joint infection (PJI) and fracture‐related infection (FRI), are among the most severe complications in orthopedic and trauma surgery. Although their incidence is relatively low, both conditions impose a disproportionate burden on patients and healthcare systems. Affected individuals frequently require multiple revision surgeries and prolonged hospitalizations. Furthermore, in cases complicated by antimicrobial resistance, an extended duration of antimicrobial therapy is often necessary, collectively contributing to substantial socioeconomic burdens [1, 2, 3, 4, 5]. Treatment complexity is further amplified by biofilm‐forming microorganisms, compromised soft tissue, and systemic comorbidities, necessitating highly individualized strategies [6, 7].

Beyond surgical challenges, implant‐associated infections have profound long‐term consequences. Patients frequently experience persistent impairments in mobility, autonomy, and reintegration into daily life. Psychological comorbidities such as anxiety and depression are also common [8, 9, 10]. Qualitative studies suggest that PJI is often perceived as a chronic condition with substantial social and emotional consequences rather than a discrete surgical complication [11, 12]. Similarly, FRI is associated with impaired fracture healing, repeated interventions, and reduced health‐related quality of life (HRQoL), sometimes comparable to that of PJI [13, 14, 15]. Registry data further confirm that HRQoL impairments may persist even after infection eradication, although the relative contribution of infection‐related effects versus pre‐existing health status remains difficult to disentangle [16, 17, 18]. Comparative studies have also highlighted differences in pathogen profiles, with staphylococci predominant but distinct distributions between PJI and FRI [19]. In addition, FRIs impose considerable health‐economic costs, largely driven by prolonged hospitalization [3].

HRQoL has therefore emerged as a key outcome domain in musculoskeletal infection research. The EQ‐5D, including its visual analogue scale (EQ‐VAS), is widely validated in orthopedic populations and provides a pragmatic, generic assessment of self‐perceived health status across functional and psychosocial dimensions, enabling comparisons across different musculoskeletal conditions and treatment strategies [20, 21, 22]. However, comparative evaluations of HRQoL between PJI and FRI remain scarce, and little is known about the influence of clinical variables such as comorbidity burden, surgical strategy, and particularly infection timing on HRQoL and length of hospital stay (LOS).

Infection timing is a long‐recognized factor influencing treatment success. In PJI, early infections are more amenable to debridement, antibiotics and implant retention (DAIR), whereas delayed or late infections are associated with biofilm maturation, lower implant retention rates and higher recurrence [23, 24, 25]. Similar trends are seen in FRI: infections within 2 weeks of fixation may be treated successfully with DAIR in nearly all cases, but success rates drop markedly after 10 weeks, often necessitating implant removal [26, 27]. Although definitions of “early,” “delayed,” and “late” vary, particularly in FRI where acute infection is often defined within weeks rather than months, the principle remains consistent: earlier intervention correlates with better outcomes, while later infections are more difficult to cure [23, 24, 25, 26, 27]. International consensus guidelines therefore recommend restricting DAIR to acute cases, typically within 4 weeks of arthroplasty or 3 weeks of symptom onset [28].

The absence of uniform diagnostic criteria has historically limited comparability between studies. While definitions such as MSIS 2011, IDSA 2013, and ICM 2018 are widely used, the more recent EBJIS 2021 definition demonstrated higher sensitivity in identifying clinically relevant infections, reflecting both progress and ongoing heterogeneity [29, 30, 31]. Registry‐based analyses also showed that reported PJI incidence can vary substantially depending on the criteria applied, underlining the urgent need for harmonization [32].

A new classification system for FRI, incorporating fracture healing status, implant presence and soft‐tissue conditions, further acknowledges the multifactorial nature of these infections [33].

Accordingly, the present study applied the widely recognized ICM/EBJIS criteria to maximize comparability [29, 34].

Management strategies are increasingly multidisciplinary. The orthoplastic (septic‐orthopedic) treatment concept, which combines bone debridement, infection control, vascular perfusion and soft‐tissue reconstruction, exemplifies the multidimensional requirements of successful therapy [35]. Within this framework, DAIR remains a cornerstone for acute PJI, but outcomes deteriorate beyond 12 weeks or in cases with sinus tracts or poor soft‐tissue conditions [23, 25, 28]. In chronic infections, radical resection of necrotic bone is essential, as devitalized tissue provides a niche for persistent biofilm [36]. Taken together, although the surgical and microbiological aspects of implant‐associated infections are well described, the impact of infection timing on HRQoL and LOS has not been systematically investigated. We therefore hypothesized that later infection timing is associated with poorer HRQoL and longer LOS in unadjusted analyses, and that these associations may differ between PJI and FRI.

This study investigated whether infection timing (early, delayed, late) is associated with HRQoL (EQ‐VAS, EQ‐5D‐3L) and length of hospital stay (LOS) in patients with implant‐associated infections. A secondary aim was to explore differences between PJIs and FRIs.

## 2 Methods

### 2.1 Study Design and Setting

This retrospective monocentric cohort study was conducted at a Level I trauma and tertiary referral center in Germany. The study was approved by the local ethics committee (reference number 23‐3468‐101). All patients had previously provided written informed consent for participation in clinical follow‐up studies and data analysis.

Patients treated for either PJI or FRI between January and December 2021 were retrospectively identified from electronic hospital records. Follow‐up was conducted 2 years later (2023) via postal questionnaire.

### 2.2 Eligibility Criteria and Infection Classification

Inclusion criteria were: age ≥ 18 years, microbiologically confirmed diagnosis of either PJI or FRI, and availability of complete clinical and follow‐up data. Exclusion criteria were culture‐negative infections, simultaneous infections at multiple sites, incomplete records, or missing follow‐up. Diagnostic classification followed internationally established criteria (MSIS 2011, ICM 2018, EBJIS 2021) [23, 28, 29, 30]. For this secondary analysis, infections were further categorized by timing as early (< 3 months after index surgery), delayed (3–24 months), or late (> 24 months), to enable direct comparison between PJI and FRI while acknowledging that earlier cut‐offs are often applied in FRI‐specific studies [23, 27].

### 2.3 Data Collection and Clinical Variables

The following demographic and clinical variables were collected: age, sex, American Society of Anesthesiologists (ASA) classification, LOS, surgical strategy (categorized as one‐stage treatment, including DAIR and one‐stage revision procedures, or two‐stage revision), reinfection, amputation, and in‐hospital mortality. Selection of surgical strategy was based on clinical judgment of the multidisciplinary treating team, considering infection chronicity, implant stability, soft‐tissue condition, and microbiological findings. Reinfection was defined as any new microbiologically confirmed infection at the same site after completion of index treatment. Revision surgery was defined as any unplanned surgical procedure related to infection management. Microbiological findings were documented for common organisms ( *Staphylococcus aureus* , *S. epidermidis* , *S. capitis* , *Enterococcus faecalis* ).

### 2.4 Patient‐Reported Outcomes

HRQoL was assessed with the EQ‐5D‐3L and EQ visual analogue scale (EQ‐VAS). The EQ‐5D‐3L evaluates five dimensions (mobility, self‐care, usual activities, pain/discomfort, anxiety/depression) on a three‐level scale, and its validity in orthopedic infection populations has been established [20, 21, 22]. EQ‐5D‐3L results are reported as the patient‐level mean across the five dimensions (range 1–3), and no utility index was calculated. In this representation, lower mean domain scores indicate better health status.

### 2.5 Statistical Analysis

Statistical analyses were performed using SPSS (version 29.0.2.0, IBM Corp., Armonk, NY, USA). Continuous variables were expressed as mean ± standard deviation (SD) and range, and compared between groups (PJI vs. FRI within each timing category) using independent‐samples *t*‐tests. Normality of continuous variables was assessed using the Shapiro–Wilk test prior to parametric analysis. Categorical variables were analyzed with Pearson's chi‐squared test or Fisher's exact test (if expected counts < 5). A two‐sided *p* value < 0.05 was considered statistically significant. Subgroup analyses, particularly for late infections, were considered exploratory due to small sample sizes. Missing data were not imputed; analyses were based on complete cases, with variable‐specific denominators reported where applicable.

**FIGURE 1:**
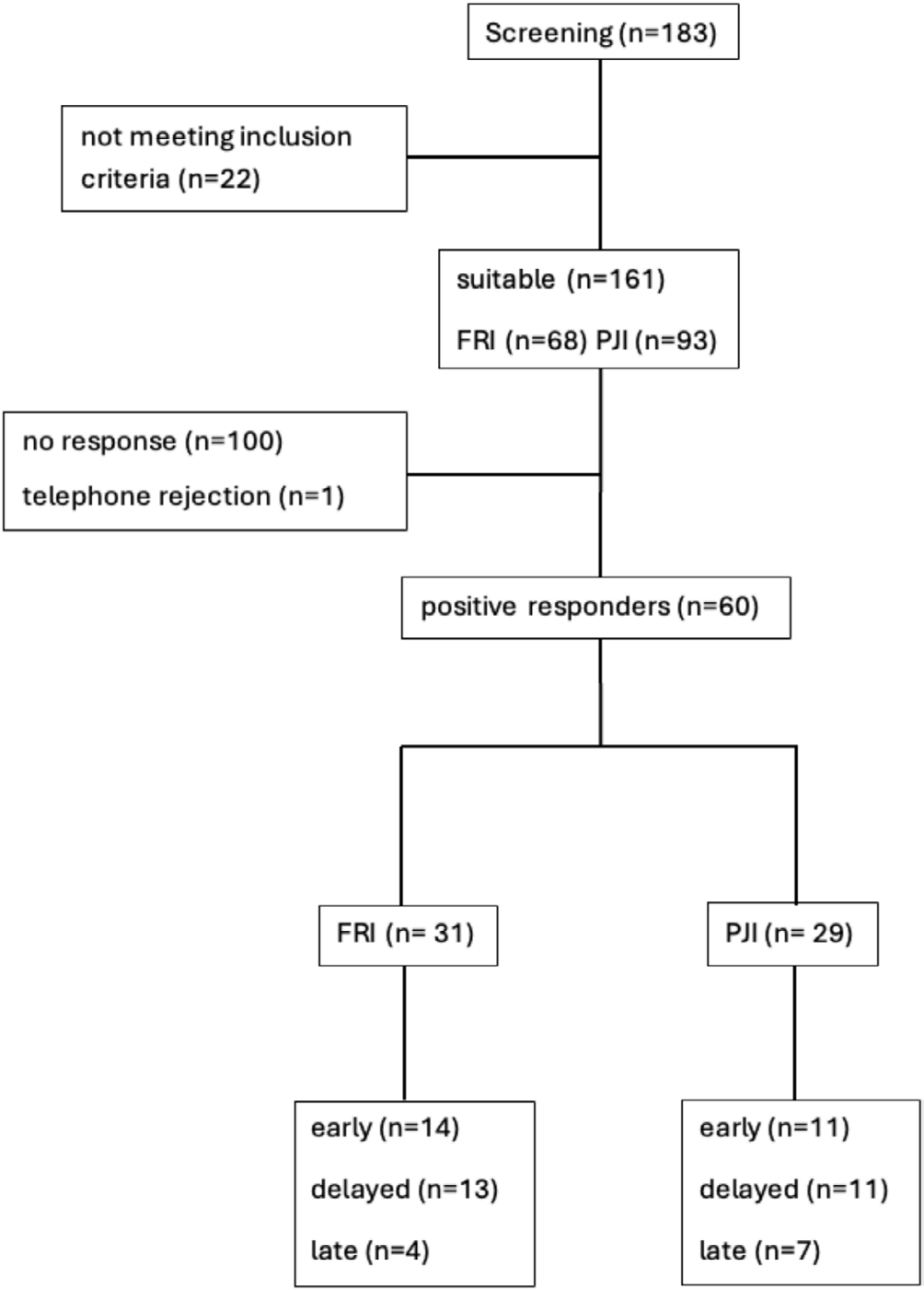
Flowchart of patient recruitment and inclusion, showing final sample of 31 FRI and 29 PJI cases stratified by infection timing (early, delayed, late).

Figure 1 illustrates the flowchart of patient screening, inclusion, and final analysis, resulting in 29 patients with PJI and 31 with FRI, stratified by infection timing (early, delayed, late).

## 3 Results

### 3.1 Patient Characteristics

A total of 60 patients were included, comprising 29 with PJI and 31 with FRI. Infections were classified as early (*n* = 25; PJI = 11, FRI = 14), delayed (*n* = 24; PJI = 11, FRI = 13), and late (*n* = 11; PJI = 7, FRI = 4). The distribution of infection timing did not differ significantly between groups (*p* = 0.507). Detailed data are presented in Table 1.

**TABLE 1:** Clinical and patient‐reported outcomes stratified by infection timing (early < 3 months, delayed 3–24 months, late > 24 months) and type of infection (periprosthetic joint infection [PJI] vs. fracture‐related infection [FRI]).

Parameter	Timing	PJI (*n* = 29)	FRI (*n* = 31)	*p*
	Early (< 3 months)	*n* = 11	*n* = 14	
EQ‐VAS		*n* = 9	*n* = 13	
Mean ± SD		48.3 ± 20.8	63.4 ± 17.8	*p* = 0.083
Min–max		25–80	20–90	
EQ‐5D‐3L		*n* = 9	*n* = 12	
Mean ± SD		1.91 ± 0.39	1.55 ± 0.33	*p* = 0.033*
Min–max		1.2–2.4	1.0–2.2	
LOS (days)		*n* = 11	*n* = 14	
Mean ± SD		39.6 ± 26.8	31.4 ± 29.0	*p* = 0.457
Min–max		5–93	8–126	
ASA classification *n* (%)		*n* = 11	*n* = 14	
I		0 (0)	1 (7.1)	
II		5 (45.5)	9 (64.2)	
III		6 (54.5)	5 (35.7)	
IV		0 (0)	0 (0)	
Reinfection		*n* = 11	*n* = 14	
*n* (%)		6 (54.5)	3 (21.4)	*p* = 0.231
Revision		*n* = 11	*n* = 14	
*n* (%)		7 (63.6)	4 (28.6)	*p* = 0.080
Amputation		*n* = 11	*n* = 14	
*n* (%)		1 (9.1)	0 (0)	*p* = 0.250
Mortality		*n* = 11	*n* = 14	
*n* (%)		1 (9.1)	1 (7.1)	*p* = 0.859
One stage treatment		*n* = 11	*n* = 14	
*n* (%)		4 (36.4)	7 (50.0)	*p* = 0.516
Main pathogens		*n* = 11	*n* = 14	
*S. epidermidis* *n* (%)		5 (45.5)	6 (42.9)	
*S. aureus* *n* (%)		5 (45.5)	4 (28.6)	
*S. faecalis* *n* (%)		1 (9.1)	4 (28.6)	
*S. capitis* *n* (%)		0 (0)	1 (7.1)	

	Delayed (3–24 months)	*n* = 11	*n* = 13	
EQ‐VAS		*n* = 10	*n* = 11	
Mean ± SD		56.6 ± 13.5	66.2 ± 16.8	*p* = 0.165
Min–max		34–80	40–95	
EQ‐5D‐3L		*n* = 8	*n* = 11	
Mean ± SD		1.76 ± 0.41	1.64 ± 0.32	*p* = 0.526
Min–max		1.0–2.0	1.0–2.2	
LOS (days)		*n* = 11	*n* = 13	
Mean ± SD		100.6 ± 82.2	28.2 ± 10.2	*p* = 0.015*
Min–max		19–233	15–49	
ASA classification *n* (%)		*n* = 11	*n* = 13	
I		0 (0)	0 (0)	
II		3 (27.3)	9 (69.2)	
III		7 (63.6)	4 (30.8)	
IV		1 (9.1)	0 (0)	
Reinfection		*n* = 11	*n* = 13	
*n* (%)		8 (72.7)	8 (61.5)	*p* = 0.493
Revision		*n* = 11	*n* = 13	
*n* (%)		6 (54.5)	12 (92.3)	*p* = 0.033*
Amputation		*n* = 11	*n* = 13	
*n* (%)		1 (9.1)	0 (0)	*p* = 0.267
Mortality		*n* = 11	*n* = 13	
*n* (%)		1 (9.1)	2 (15.4)	*p* = 0.642
One stage treatment		*n* = 11	*n* = 13	
*n* (%)		3 (27.3)	2 (15.4)	*p* = 0.496
Main pathogens		*n* = 11	*n* = 13	
*S. epidermidis* *n* (%)		7 (63.6)	4 (30.8)	
*S. aureus* *n* (%)		3 (27.3)	9 (69.2)	
*S. faecalis* *n* (%)		3 (27.3)	3 (23.1)	
*S. capitis* *n* (%)		0 (0)	3 (23.1)	

	Late (> 24 months)	*n* = 7	*n* = 4	
EQ‐VAS		*n* = 6	*n* = 3	
Mean ± SD		43.3 ± 23.4	39.7 ± 25.9	*p* = 0.847
Min–max		10–76	20–69	
EQ‐5D‐3L		*n* = 5	*n* = 3	
Mean ± SD		1.80 ± 0.40	1.55 ± 0.64	*p* = 0.479
Min–max		1.4–2.4	1.4–2.6	
LOS (days)		*n* = 7	*n* = 4	
Mean ± SD		115.9 ± 118.0	55.3 ± 44.5	*p* = 0.257
Min–max		15–341	20–120	
ASA classification *n* (%)		*n* = 7	*n* = 4	
I		0 (0)	1 (25.0)	
II		2 (28.6)	1 (25.0)	
III		5 (71.4)	2 (50.0)	
IV		0 (0)	0 (0)	
Reinfection		*n* = 7	*n* = 4	
*n* (%)		5 (71.4)	4 (100.0)	*p* = 0.657
Revision		*n* = 7	*n* = 4	
*n* (%)		1 (14.3)	1 (25.0)	*p* = 0.554
Amputation		*n* = 7	*n* = 4	
*n* (%)		0 (0)	0 (0)	*p* = 1.000
Mortality		*n* = 7	*n* = 4	
*n* (%)		0 (0)	0 (0)	*p* = 1.000
One stage treatment		*n* = 7	*n* = 4	
*n* (%)		0 (0)	0 (0)	*p* = 1.000
Main pathogens				
*S. epidermidis* *n* (%)		6 (85.7)	2 (50)	
*S. aureus* *n* (%)		2 (28.6)	2 (50)	
*S. faecalis* *n* (%)		1 (14.3)	0 (0)	
*S. capitis* *n* (%)		2 (28.6)	0 (0)	

*Note:* Data are presented as mean ± standard deviation (SD) with range, or number (percentage). One‐stage treatment includes debridement, antibiotics and implant retention (DAIR) as well as one‐stage revision procedures. Comparisons in the late infection subgroup are presented descriptively due to very small sample sizes; *p* values are shown for completeness but should not be interpreted inferentially. *p* values refer to comparisons between PJI and FRI within each timing category. *p < 0.05 indicates statistical significance.

### 3.2 Patient‐Reported Outcomes

**FIGURE 2:**
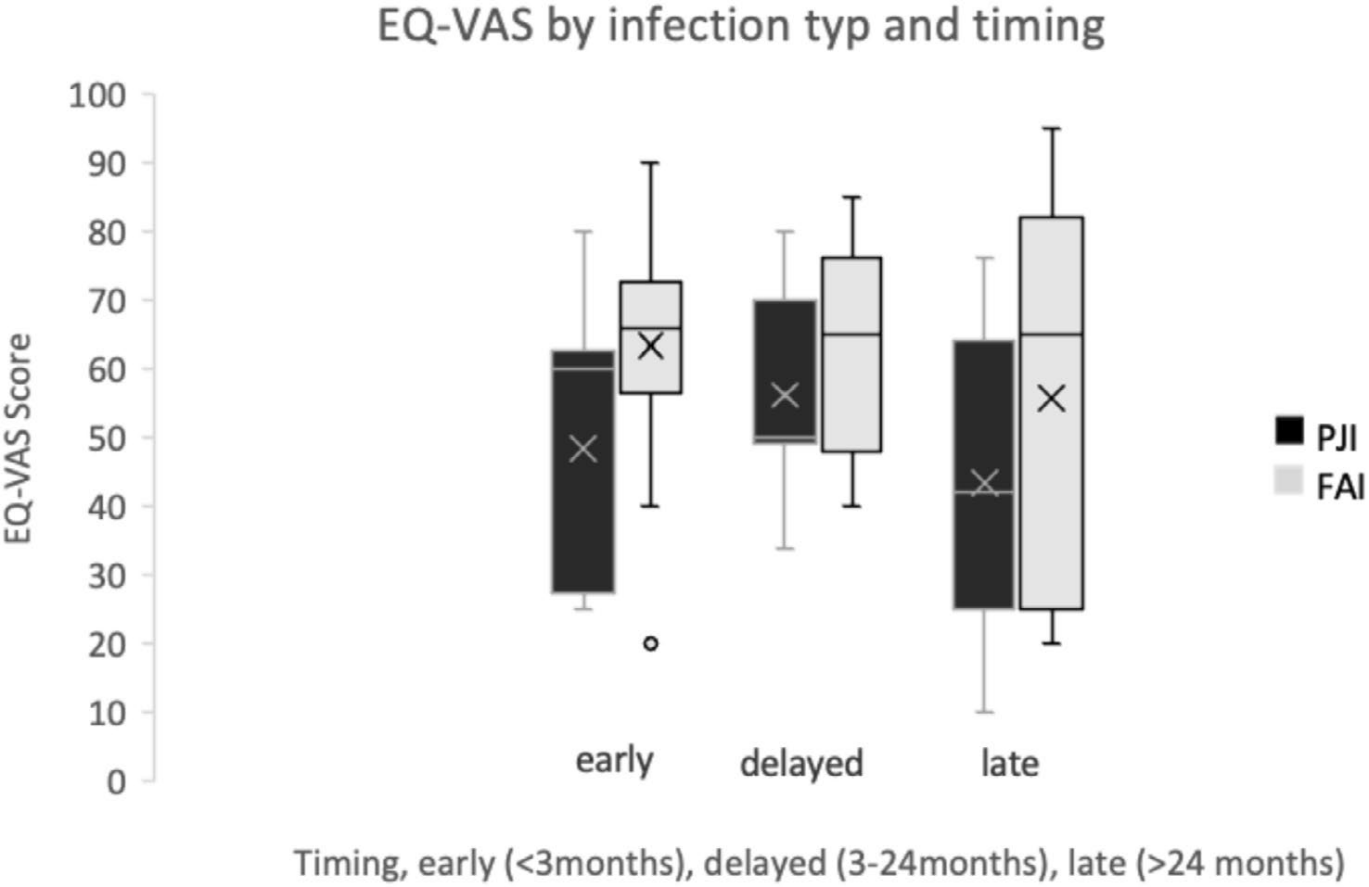
Boxplot of EQ‐VAS scores stratified by infection timing and infection type (PJI vs. FRI).

EQ‐VAS scores were consistently higher in FRI than in PJI for early (63.4 ± 17.8 vs. 48.3 ± 20.8; *p* = 0.083) and delayed infections (66.2 ± 16.8 vs. 56.6 ± 13.5; *p* = 0.165) in unadjusted, exploratory analyses, while both groups showed marked declines in late infections (39.7 ± 25.9 vs. 43.3 ± 23.4; analyzed descriptively due to very small subgroup sizes). Confidence intervals illustrated the uncertainty of these estimates, particularly in the smaller subgroups: early PJI 48.3 (95% CI 34.7–61.9) versus early FRI 63.4 (95% CI 53.0–73.8); delayed PJI 56.6 (95% CI 47.6–65.6) versus delayed FRI 66.2 (95% CI 55.3–77.1); late PJI 43.3 (95% CI 21.5–65.2) versus late FRI 39.7 (95% CI 5.0–74.3) (Figure 2).

The EQ‐5D‐3L mean domain score was consistent with these findings. In unadjusted analyses, scores were significantly lower in early PJI compared with FRI (1.91 ± 0.39 vs. 1.55 ± 0.33; *p* = 0.033), with the largest difference in the anxiety/depression dimension (*χ* ^2^ = 6.24, *p* = 0.044). No significant differences were found in delayed or late infections.

### 3.3 Length of Stay

**FIGURE 3:**
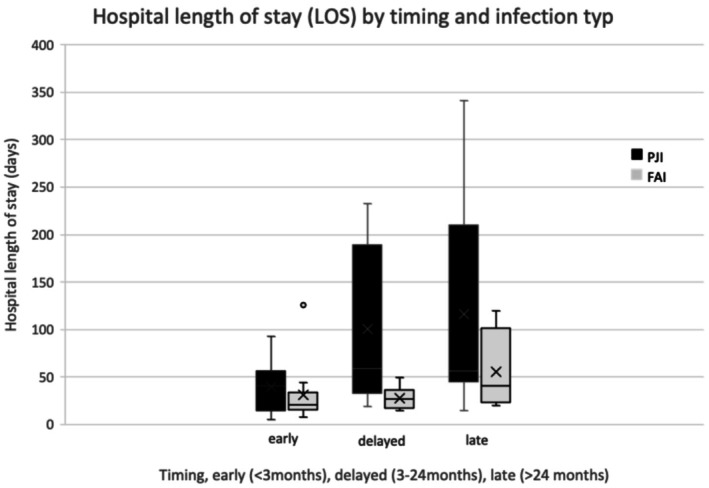
Length of hospital stay (LOS) by infection timing and infection type. Boxplots show median, interquartile range, and outliers.

LOS increased with infection chronicity, particularly in PJI (early 39.6 ± 26.8 days vs. 31.4 ± 29.0; delayed 100.6 ± 82.2 vs. 28.2 ± 10.2; late 115.9 ± 118.0 vs. 55.3 ± 44.5). The difference was significant in delayed infections (*p* = 0.015) (Figure 3).

### 3.4 Clinical Outcomes

Reinfection rates appeared to increase with later infection timing in both groups (PJI: 54.5% early; 72.7% delayed; 71.4% late; FRI: 21.4%, 61.5%, 100%). Revision patterns diverged: early infections required more revisions in PJI (63.6% vs. 28.6%, *p* = 0.080), whereas delayed infections had significantly higher revision rates in FRI (92.3% vs. 54.5%, *p* = 0.033).

With regard to surgical strategy, one‐stage treatment (including DAIR and one‐stage revision procedures) was more frequent in early infections (PJI 4/11; FRI 7/14) than in delayed infections (PJI 3/11; FRI 2/13) and was not performed in late infections. The remaining cases were managed with two‐stage revision procedures. Detailed timing‐stratified data are presented in Table 1.

Amputation (≤ 9%) and in‐hospital mortality (≤ 15%) were rare and showed no significant differences between PJI and FRI.

### 3.5 Microbiological Findings

Staphylococci were the most frequent pathogens across groups. In early infections, *S. epidermidis* and *S. aureus* were predominant. In delayed infections, *S. aureus* dominated in FRI (69.2%) while *S. epidermidis* was more common in PJI (63.6%; *p* = 0.041). Late PJIs were mainly caused by coagulase‐negative staphylococci ( *S. epidermidis* 85.7%), whereas FRIs showed a mixed pattern.

## 4 Discussion

### 4.1 Principal Findings

This study examined associations between infection timing and outcomes in patients with PJI and FRI. Our results illustrate the clinical relevance of stratifying infections into early, delayed, and late phases, as this classification was associated with differences in treatment strategies and patient‐reported outcomes. Later infections were associated with longer hospitalization, higher reinfection rates, and poorer HRQoL in unadjusted analyses, while revision patterns differed between PJI and FRI.

The importance of infection timing has long been recognized. Zimmerli et al. established the widely accepted temporal classification for PJI (< 3 months, 3–24 months, > 24 months), which has since been applied to FRI by Kuehl and Morgenstern [23, 26, 27]. Early infections are more likely to respond to DAIR, whereas delayed and late infections typically require staged procedures with higher morbidity [24, 25, 27]. Our findings are consistent with this body of literature, demonstrating increasing reinfection and revision rates with advancing infection chronicity.

### 4.2 Differences Between PJI and FRI

A notable finding was the divergent revision pattern between entities: early infections were revised more frequently in PJI, whereas delayed infections required significantly more revisions in FRI. This difference may reflect distinct anatomical and surgical contexts. Early PJI is often managed with DAIR, whereas delayed FRI frequently occurs in compromised bone and soft tissue conditions, where implant stability is reduced and staged reconstruction becomes necessary [2, 26, 33]. These findings suggest the need for entity‐specific treatment algorithms and are in line with the growing consensus that PJI and FRI, despite shared microbiological features, should be regarded as distinct clinical entities [2, 14, 33].

### 4.3 Patient‐Reported Outcomes and Microbiological Findings

Patient‐reported outcomes added a meaningful perspective. EQ‐VAS and EQ‐5D scores declined markedly in late infections, particularly in PJI, in unadjusted analyses, reflecting the long‐term functional and psychosocial burden previously described [9, 10, 13, 16, 17]. Consistent with prior studies, many patients continued to experience reduced mobility, persistent pain, and psychological distress even after infection control [11, 12]. Interestingly, FRI patients in this cohort reported higher EQ‐VAS scores than PJI patients in early and delayed infections. This may reflect differences in baseline health and functional reserve (e.g., generally younger age and better pre‐traumatic physical function in patients with FRI), patient expectations, or the impact of prosthesis loss on daily functioning [3, 13, 15, 16]. This observation is consistent with a previous analysis of the same cohort, which demonstrated lower HRQoL in PJI compared with FRI and suggested that this difference was partly mediated by a higher comorbidity burden and longer hospital stay rather than infection entity alone [37].

The microbiological profiles were in line with existing literature, with staphylococci predominating across all groups [2, 14, 26]. In delayed FRI, *Staphylococcus aureus* was markedly more common, consistent with its more aggressive course and the higher revision rates observed [2, 26]. Conversely, late PJI was mainly associated with coagulase‐negative staphylococci. These findings are consistent with international data on FRIs, suggesting that pathogen‐specific characteristics may influence revision rates and outcomes [38].

### 4.4 International Context and Clinical Implications

Our results should also be considered in a broader international context. Most data on PJI and FRI originate from Europe and North America, but recent registry studies from Australasia and North America have demonstrated significant differences in treatment strategies, particularly regarding the use of DAIR versus staged procedures [34]. Furthermore, evidence from low‐ and middle‐income countries (LMICs) reveals unique challenges, including limited diagnostic resources, delayed referral, and greater dependence on suppressive antibiotic therapy [39]. These systemic factors often result in poorer outcomes than those seen in high‐income healthcare systems. Although our study was conducted in a high‐resource tertiary center, the observed trends, especially the importance of early diagnosis and timely intervention, may be applicable more widely.

### 4.5 Strengths and Limitations

This study has several strengths. First, it enables a direct comparison of PJIs and FRIs within a single cohort, employing the same methodological framework. Second, the study systematically stratified infections according to established timing categories (early, delayed, and late), enabling the exploration of differences in clinical outcomes and patient‐reported quality of life according to timing. Third, the inclusion of validated patient‐reported outcome measures (EQ‐VAS and EQ‐5D‐3L) provides an important patient‐centered perspective, which is frequently overlooked in musculoskeletal infection research.

However, this study has several limitations. The retrospective, single‐center design and limited sample size restrict statistical power, particularly with regard to late infections. The cohort reflects a tertiary referral population, which introduces potential selection bias towards complex or treatment‐resistant cases. Surgical strategies and follow‐up intervals were heterogeneous, and treatment decisions were made individually based on multidisciplinary clinical judgment considering infection chronicity, implant stability, soft‐tissue condition and microbiological findings. This may have influenced revision rates and other clinical outcomes. Baseline HRQoL data prior to infection were unavailable, limiting the interpretation of patient‐reported outcomes. Furthermore, outcome variability may arise from differing diagnostic definitions. Previous research has shown that reported PJI rates and outcomes can vary substantially depending on the criteria applied, with discrepancies between the EBJIS, ICM and IDSA definitions [29, 34]. Recent multicenter analyses confirmed that these definitional inconsistencies directly affect reported infection rates and highlight the need for global harmonization [34].

Although we used ICM/EBJIS criteria to ensure comparability, this heterogeneity remains a potential source of bias.

Future research should prioritize early detection strategies for musculoskeletal infections, including biomarker development, molecular imaging, and artificial intelligence–based risk stratification [36, 40]. Prospective multicenter studies are warranted to validate our findings and to determine whether timing‐specific treatment pathways improve outcomes. Moreover, the routine integration of patient‐reported measures into infection management research is essential to capture the long‐term impact of these conditions on patients' lives. Overall, our findings suggest that infection timing is an important factor associated with clinical and patient‐centered outcomes and support international efforts to standardize definitions and optimize treatment protocols.

## 5 Conclusion

Infection timing was associated with both clinical and patient‐reported outcomes in implant‐associated infections. Earlier infections were generally linked to better quality of life and shorter hospital stays, whereas delayed and late infections were associated with higher reinfection and revision rates in unadjusted analyses. Distinct patterns between PJIs and FRIs underline the need for tailored, entity‐specific management strategies. These findings highlight the importance of harmonized diagnostic standards and multidisciplinary treatment approaches to improve long‐term outcomes.

## Author Contributions

Conceptualization: Jasper Frese, Arndt‐Peter Schulz. Methodology: Jasper Frese, Arndt‐Peter Schulz. Investigation: Lena Schwake. Data curation: Jasper Frese, Sidney Schaeffer, Arndt‐Peter Schulz. Formal analysis: Lena Schwake, Sidney Schaeffer. Writing – original draft: Lena Schwake. Writing – review and editing: Jasper Frese, Arndt‐Peter Schulz, Sidney Schaeffer, Ulf‐Joachim Gerlach, Cornelius Grimme. Supervision: Jasper Frese, Arndt‐Peter Schulz, Ulf‐Joachim Gerlach, Cornelius Grimme.

## Funding

The authors have nothing to report.

## Disclosure

All authors meet the criteria for authorship as defined by the International Committee of Medical Journal Editors (ICMJE). All authors have approved the final version of the manuscript and agree to be accountable for all aspects of the work.

## Ethics Statement

This study was conducted in accordance with the principles of the Declaration of Helsinki and approved by the Ethics Committee of the University of Regensburg (reference number 23‐3468‐101). All participants provided written informed consent prior to inclusion.

## Conflicts of Interest

The authors declare no conflicts of interest.

## Supporting information


**Data S1:** Validated German version of EQ‐5D‐3L and EQ‐VAS questionnaire (EuroQol Group 2009).

## Data Availability

The data that support the findings of this study are available on request from the corresponding author. The data are not publicly available due to privacy or ethical restrictions.
