# Supplementary material for: Impact of Infection Timing on Outcomes: A Comparative Study of Periprosthetic Joint and Fracture‐Related Infections
Source: Orthop Surg. 2026 Apr 21;18(6):1173–82. doi: 10.1111/os.70323 (PMC13238746; doi:10.1111/os.70323)

**Supplementary Material 1:** validated German version of EQ-5D-3L and EQ-VAS questionnaire (EuroQol Group 2009):


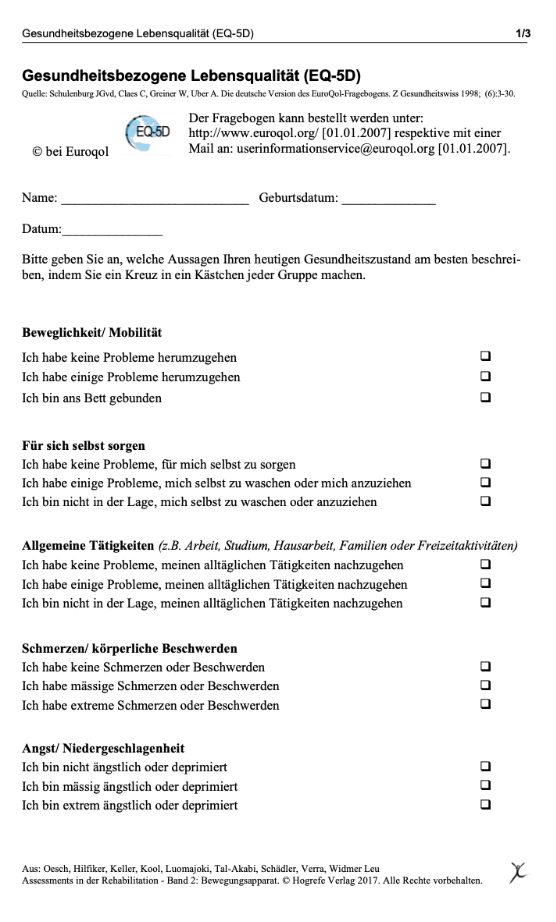

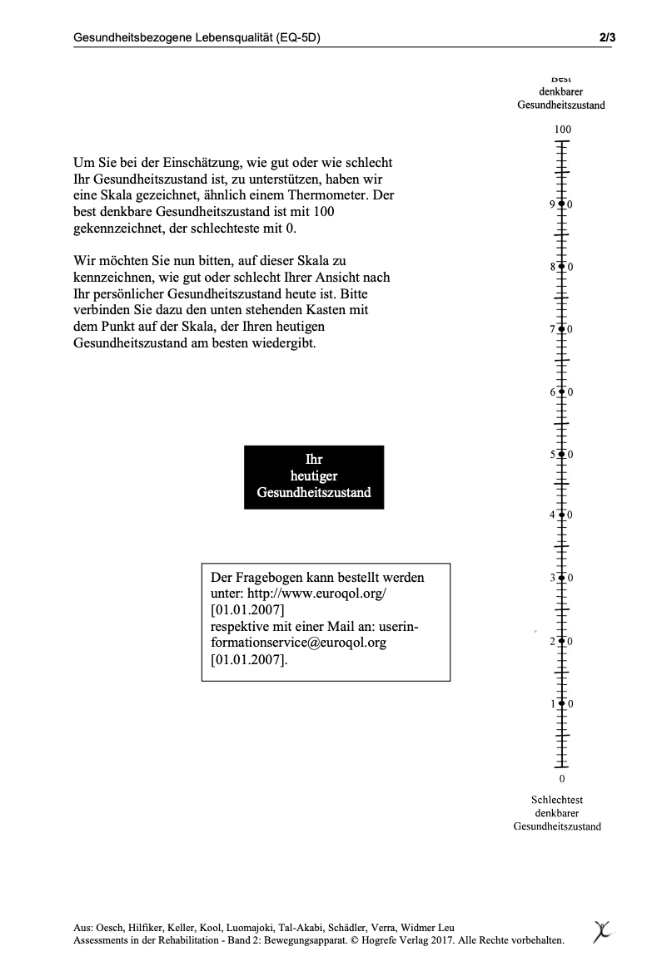

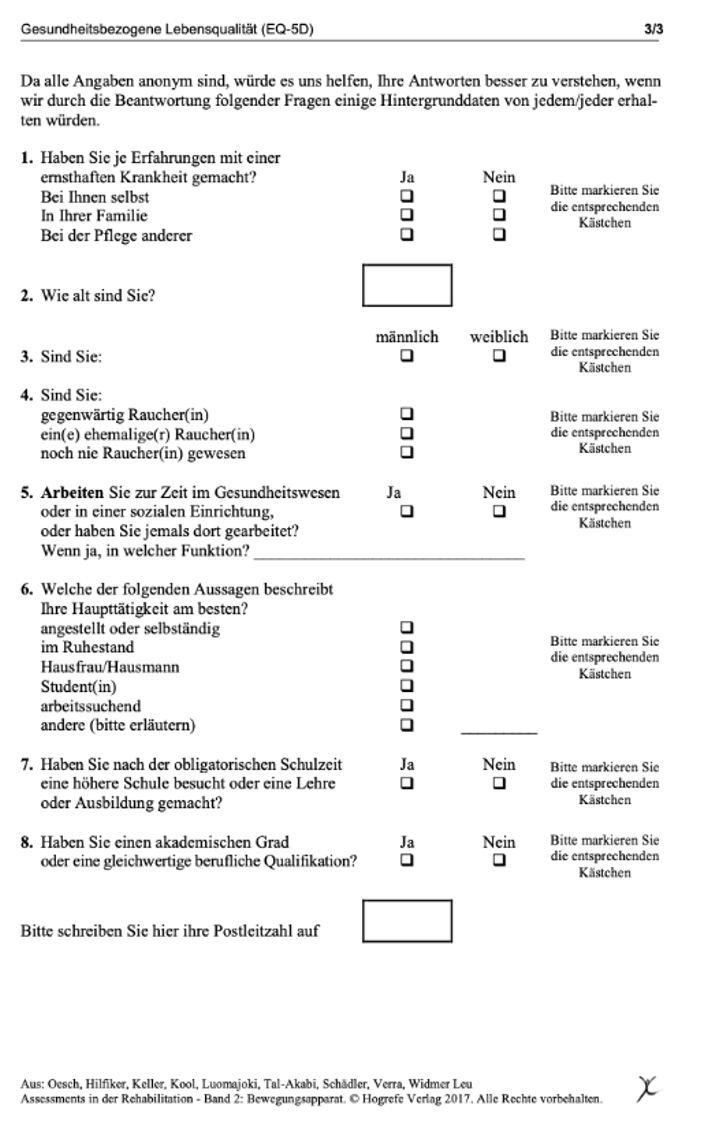

Supplement: Supplementary file 1 — Data S1: Validated German version of EQ‐5D‐3L and EQ‐VAS questionnaire (EuroQol Group 2009). [file OS-18-1173-s001.docx]
